# Predicting U.S. county opioid poisoning mortality from multi-modal social media and psychological self-report data

**DOI:** 10.1038/s41598-023-34468-2

**Published:** 2023-06-03

**Authors:** Salvatore Giorgi, David B. Yaden, Johannes C. Eichstaedt, Lyle H. Ungar, H. Andrew Schwartz, Amy Kwarteng, Brenda Curtis

**Affiliations:** 1 National Institute on Drug Abuse, Intramural Research Program, Baltimore, MD USA; 2 Department of Computer and Information Science, University of Pennsylvania, Philadelphia, PA USA; 3 Department of Psychiatry and Behavioral Sciences, Johns Hopkins University School of Medicine, Baltimore, MD USA; 4 Department of Psychology, Stanford University, Stanford, CA USA; 5 Institute for Human-Centered AI, Stanford University, Stanford, CA USA; 6 Department of Computer Science, Stony Brook University, Stony Brook, NY USA

**Keywords:** Risk factors, Public health

## Abstract

Opioid poisoning mortality is a substantial public health crisis in the United States, with opioids involved in approximately 75% of the nearly 1 million drug related deaths since 1999. Research suggests that the epidemic is driven by both over-prescribing and social and psychological determinants such as economic stability, hopelessness, and isolation. Hindering this research is a lack of measurements of these social and psychological constructs at fine-grained spatial and temporal resolutions. To address this issue, we use a multi-modal data set consisting of natural language from Twitter, psychometric self-reports of depression and well-being, and traditional area-based measures of socio-demographics and health-related risk factors. Unlike previous work using social media data, we do not rely on opioid or substance related keywords to track community poisonings. Instead, we leverage a large, open vocabulary of thousands of words in order to fully characterize communities suffering from opioid poisoning, using a sample of 1.5 billion tweets from 6 million U.S. county mapped Twitter users. Results show that Twitter language predicted opioid poisoning mortality better than factors relating to socio-demographics, access to healthcare, physical pain, and psychological well-being. Additionally, risk factors revealed by the Twitter language analysis included negative emotions, discussions of long work hours, and boredom, whereas protective factors included resilience, travel/leisure, and positive emotions, dovetailing with results from the psychometric self-report data. The results show that natural language from public social media can be used as a surveillance tool for both predicting community opioid poisonings and understanding the dynamic social and psychological nature of the epidemic.

## Introduction

Opioid poisoning mortality (OPM; see the Ethics Statement below for a description on the use of “poisoning” vs. the term “overdose”), both intentional and unintentional, is a substantial public health issue that has received national policy attention. In 2020, there were 91,799 fatal drug poisonings in the United States (U.S.) and 75% were due to opioids^1^. It is now impacting a higher proportion of the population and a more diverse set of communities: while initially thought to be limited to mostly rural and White demographics, the impacts on urban and African American populations has been under reported^2,3^. Indeed, the rate of fatal drug poisonings increased during the years between 1999-2017 across every demographic group and in every geographical region of the U.S.^4^, with the COVID-19 pandemic^5–7^ and increased availability of fentanyl and other synthetic opioids^7,8^ contributing to more recent increases. To coincide with the availability of various opioids, the Centers for Disease Control and Prevention (CDC) defined three waves across the opioid epidemic: (1) a rise in prescription opioid poisonings starting in 1999, (2) a rise in heroin poisonings starting in 2010, and (3) a rise in synthetic opioid poisonings starting in 2013^9^.

Considerable attention has been given to the supply side of the opioid epidemic, with over-prescribing by both physicians and pharmaceutical companies thought to drive mortality rates^10^. While prescribing is certainly a factor in opioid availability, it ignores root causes and potential structural factors which lead to increased demand. As such, several studies have investigated both physical and psychological determinants, arguing that the epidemic is driven by economic and social upheaval, including trauma, hopelessness, and disadvantage^11^. Communities experiencing increased OPM have been characterized by low subjective well-being^12–14^, education and income inequality^15–17^, low social capital^18^, family distress^19^, low healthcare quality^20^, and food insecurity^21^.

However, much of the available social, psychological, and economic data is limited both spatially and temporally. For example, data from the CDC’s 2021 Behavioral Risk Factor Surveillance System survey, one of the largest continuous health surveys, contains roughly 450,000 responses, which may not have the proper data density to obtain stable sub-state or sub-annual measurements across the entire U.S. There is also considerable lag in public release of both the structural and OPM data, e.g., the U.S. Census occurs every ten years and the release of annual CDC OPM rates is delayed around 11 months after the start of the calendar year^22^. These data issues have not gone unnoticed at the local and federal levels. Starting in 2020, the CDC’s National Vital Statistics System expanded provisional data releases in an attempt to deliver near real-time mortality rates^22^. More recently, to improve outbreak response in collaboration with state and local governments, the CDC has implemented the “OverdoseData2Action” plan^23^ and launched the Center for Forecasting and Outbreak Analytics^24^. Finally, many constructs of interest may not be available in a form suitable for large-scale spatial analysis. For example, hopelessness and loneliness have been shown to be associated with individual-level substance use^25^ and are also thought to be potential structural factors related to the opioid epidemic^11^. To the best of our knowledge, no large national studies track either construct.

As such, several studies have introduced methodological frameworks for the prediction of OPM, which include multi-modal data sources^26^ and machine learning based approaches^27^. One possible solution in line with these calls, is to leverage large, public data sets of natural language from social media, such as Twitter and Reddit, alongside traditional data sources. Social media data has been widely used in population health studies, including forecasting, pharmacovigilance, and surveillance applications^28,29^, but also in the domains of mental health and psychology^30–32^. Often containing billions of individual data points, social media language allows for both fine-grained temporal^33^ and spatial^34^ analysis. In the domain of OPM, the typical application is to measure the frequency of opioid related keywords (e.g., mentions of the word “opioid” or “fentanyl”) in order to track mortality rates or prescriptions^35–40^. While such keyword approaches can accurately predict real-world outcomes, they may fail to fully characterize communities, analogous to examining over-prescribing.

This study tests the feasibility of using machine-learning-based, natural language processing models to identify the primary structural predictors of OPM. In particular, we used multi-modal data sources (i.e., both national surveys and publicly available social media data) to characterize U.S. county-level OPM and predict future mortality across waves of the opioid epidemic (e.g., increases in heroin or synthetic opioid poisonings). Unlike previous studies that have used natural language processing algorithms and social media data, we do not focus on opioid or drug-related keywords. Instead, we focused on data driven open-vocabulary approaches, which do no rely on a priori hypotheses on which words should be related to OPM. The data driven nature of these methods allows one to gain insights into the types of communities suffering from a given outcome (in our case OPM) and has been used to study a diverse set of community outcomes, including subjective well-being, substance use, and mortality^41–45^. We compared the language-based predictors against several factors which have been identified as possible explanations of the mortality increase^46^, including psychological well-being, socio-demographics, deficiencies in health care, and forms of inequality.

We proceed in three steps using several heterogeneous data sets, which included self-reports, census data, and publicly available social media data (i.e., Twitter): (1) establish a predictive baseline using psychometric self-reports and socio-demographic variables known to be associated with OPM, (2) establish Twitter-based language measures to predict OPM at levels comparable to the psychometric self-reports, and (3) further understand the extent to which Twitter predicts mortality by examining the most predictive words and categories of words. We conclude by examining how these factors relate to temporal trends in OPM.

We show that U.S. counties suffering from high OPM can be characterized by markers of negative emotions, lack of access to healthcare, and increased physical pain, as revealed by both psychometric self-reports and community-level language. Notably, the most predictive Twitter words are *not* related to opioids or substance use. The results support the view that, in addition to over prescribing, the opioid epidemic is driven by economic and social disadvantage^11^ and natural language from social media can provide a lens into the physical, emotional, and psychological distress of a community.

## Results

### Predictive baseline via psychometric self-reports and area-based covariates

**Figure 1:**
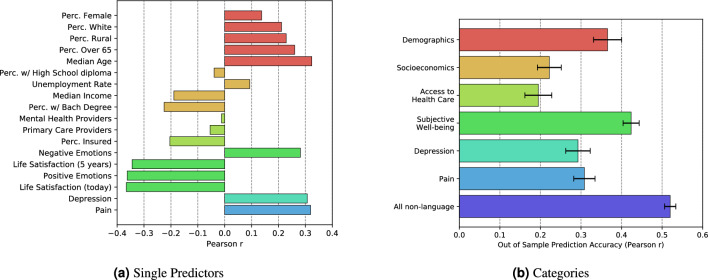
Correlations with opioid mortality. Panel (**a**) shows the Pearson correlation between each variable and OPM, ordered within their respective categories by ascending effect size. Panel (**b**) shows the out-of-sample, cross-validated Pearson r (standard error computed across the tenfolds) for each category.

Figure 1a shows the Pearson correlation between each variables and OPM. Here we see the variables most associated with increased OPM were low life satisfaction, low positive emotions, higher median age, increased physical pain, and increased depression. Next, we combine all predictors into higher level categories (e.g., life satisfaction and positive/negative emotions into subjective well-being) and evaluate the out-of-sample prediction accuracy of the category as a whole. Figure 1b shows that subjective well-being has the highest prediction accuracy ($r=0.42$), followed by demographics ($r=0.37$). Notably, the prediction accuracies of the demographics and subjective well-being categories are higher than that of any single predictor within each category, whereas this is not the case for socioeconmics or access to healthcare. This suggests that multiple variables within the demographics and subjective well-being categories are independently contributing to the category’s accuracy, whereas the categories are mostly driven by a single variable. We note that similar patterns hold when controlling for confounding variables – that is, the physical and psychological well-being measures remained significantly associated with mortality rates when controlling for all other categories of variables (see Supplementary Tables S6 through S11). Finally, combining all psychometric self-reports and area-based covariates together into a single category gives the highest prediction accuracy ($r=0.52$).

**Figure 2:**
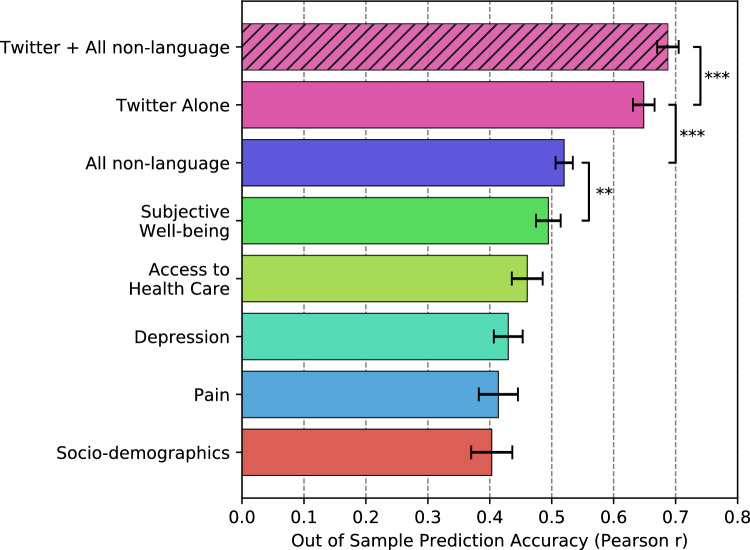
Predicting opioid mortality, reported out of sample prediction accuracy (Pearson r) from tenfold cross validation (standard error computed across the tenfolds). Each model (except Twitter alone) contains demographics and socio-economics to show the predictive contribution of each set of variables above standard socio-demographic measures. ***${{p}} < 0.001$, ** ${{p}} < 0.01$ paired t-test between the absolute error of each model.

### Predictive accuracy of Twitter

Figure 2 compares the out-of-sample predictive accuracy of the psychometric self-reports and area-based covariates to the Twitter-based machine learning model. Here, all models contained both demographics and socio-economics as covariates in order to understand the contribution of each category of variables above and beyond standard socio-demographic measures. The “All non-language” line contained all non-Twitter variables and the “Twitter + All non-language” line contained all psychometric self-reports, area-based covariates, and Twitter language features. In terms of the psychometric self-reports and area-based covariates, the Subjective Well-being category had the highest out of sample prediction accuracy ($r=0.49$), above that of access to healthcare ($r=0.46$), depression ($r=0.43$), and physical pain ($r=0.41$). The Subjective Well-being model’s accuracy was also statistically higher than that of the next best performing model, Access to Health Care. Next, the Twitter model ($r=0.65$) outperformed all other models and was statistically different from the “All non-language” model ($t=4.75$, $p<0.001$). Adding everything together in a single model, we see that the “Twitter + All non-language” model had higher out-of-sample prediction accuracy ($r=0.68$) than ‘Twitter Alone” ($t=4.39$, $p<0.001$), showing that Twitter, the psychometric self-reports, and the area-based covariates all contribute unique signal when predicting OPM. Supplemental Materials Table S12 shows that “Twitter + All non-language” model is able to predict OPM within 7.1 age-adjusted deaths per 100,000 people (on average), while the “All non-language” model predicts within 8.5 adjusted deaths per 100,000 people (on average).

**Figure 3:**
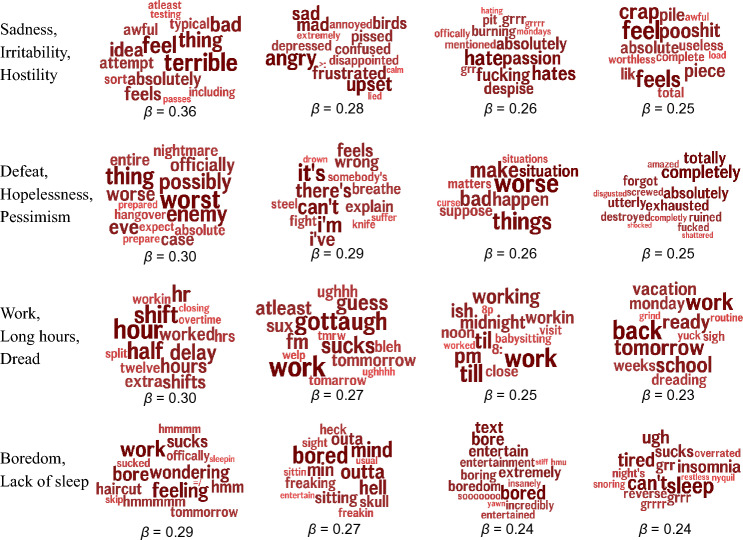
Topics positively correlated with opioid mortality rates; higher rates of topic usage associated with higher mortality. Reported standardized betas. All correlations are significant at a Benjamini–Hochberg corrected significance threshold of ${{p}} < 0.05$ with socio-demographics added as covariates.

### Linguistic insights

**Figure 4:**
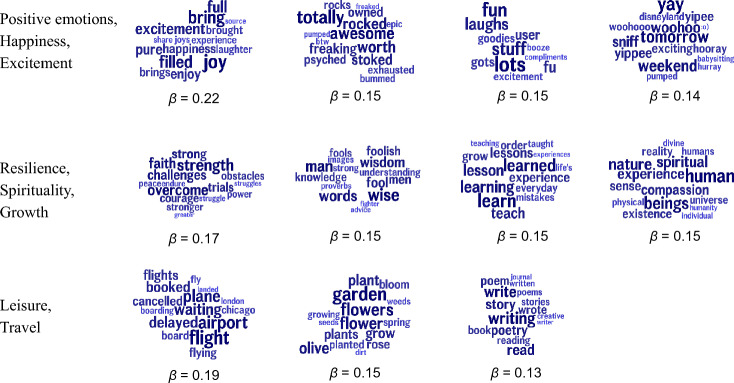
Topics negative correlated with opioid mortality rates; higher rates of topic usage associated with lower mortality. Reported standardized betas. All correlations are significant at a Benjamini-Hochberg corrected significance threshold of ${{p}} < 0.05$ with socio-demographics added as covariates.

The linguistic predictors of OPM can be interpreted to some extent. Figure 3 shows the topics positively correlated with OPM (i.e., counties which use these topics more frequently experience higher OPM). Overall themes, which were manually labeled, include sadness (*feel*, *sad*, *terrible*), hopelessness (*worst*, *utterly*, *can’t*), work/working long hours/dreading work (*shifts*, *dreading*, *grind*), and boredom (*bore*, *sitting*, *hmu*). Figure 4 shows the topics negatively correlated with OPM (i.e., counties which use these topics more frequently experience lower OPM). Here we see positive emotions (*joy*, *awesome*, *woohoo*), growth/spirituality (*overcome*, *learned*, *spiritual*) and leisure/travel (*airport*, *garden*, *poetry*).

### Temporal trends in OPM

Finally, we examine how the psychometric self-reports, social media language, and area-based covariates predict future changes in OPM, as defined by the three waves of the opioid epidemic outlined by the CDC. Table 1 shows how these items, measured during Wave 2, were related to both future mortality rates (i.e., Wave 3 mortality) and changes in mortality rates (i.e., the difference between Wave 3 and Wave 2 mortality rates). The first column (Wave 3 Mortality) is consistent with the above results (Fig. 1a) in that higher future mortality during Wave 3 was associated with lower positive emotions, life satisfaction, percentage of population insured, median income, and college education. Higher future mortality during Wave 3 was also associated with higher levels of depression, physical pain, negative emotions, and demographics. In the second column $\Delta$(Wave 3–Wave 2), lower life satisfaction, positive emotions, and insurance rates were associated with an increased wave-to-wave *change* in mortality (i.e., rates during Wave 3 are higher than in Wave 2). Of note, depression, physical pain, income, education, percent rural, and percent white were not significantly associated with changes in mortality across the two waves, despite being significantly associated with higher mortality during Wave 3 alone. Finally, we see that Twitter has a higher out-of-sample prediction accuracy when compared to all psychometric self-reports and area-based covariates. This is true for both future mortality (Wave 3) and changes in mortality from Wave 2 to Wave 3.

**Table 1:** Correlates of future OPM waves.

	Wave 3 Mortality	$\Delta$(Wave 3–Wave 2)
Demographics		
Perc. female	0.19 [0.11, 0.27]***	0.24 [0.16, 0.32]***
Perc. white	0.21 [0.12, 0.29]***	0.06 [−0.03, 0.14]
Perc. rural	0.16 [0.08, 0.24]***	0.06 [−0.03, 0.14]
Perc. over 65	0.25 [0.16, 0.32]***	0.12 [0.03, 0.20]**
Median age	0.31 [0.24, 0.39]***	0.23 [0.15, 0.31]***
Socioeconomics		
Perc. w/High School Diploma	0.04 [−0.04, 0.13]	0.08 [−0.00, 0.17]
Unemployment Rate	0.05 [−0.04, 0.13]	0.02 [−0.06, 0.11]
Median Income	−0.13 [−0.21, −0.04]**	0.01 [−0.08, 0.10]
Perc. w/Bachelor’s Degree	−0.15 [−0.24, −0.07]***	−0.02 [−0.11, 0.07]
Access to Healthcare		
Mental Health Providers	−0.02 [−0.11, 0.07]	−0.00 [−0.09, 0.09]
Primary Care Providers	−0.02 [−0.10, 0.07]	0.03 [−0.06, 0.11]
Perc. Insured	−0.29 [−0.37, −0.21]***	−0.36 [−0.43, −0.28]***
Subjective well-being		
Positive Emotions	−0.32 [−0.39, −0.24]***	−0.25 [−0.33, −0.17]***
Negative Emotions	0.21 [0.12, 0.29]***	0.11 [0.02, 0.19]*
Life Satisfaction (today)	−0.32 [−0.39, −0.24]***	−0.20 [−0.28, −0.12]***
Life Satisfaction (5 years)	−0.27 [−0.35, −0.19]***	−0.10 [−0.18, −0.01]*
Depression	0.22 [0.13, 0.30]***	0.02 [−0.06, 0.11]
Pain	0.21 [0.12, 0.29]***	−0.02 [−0.10, 0.07]
All$^{\dagger }$	0.53 [0.46, 0.58]***	0.51 [0.43, 0.57]***
Twitter$^{\dagger }$	0.59 [0.53, 0.65]***	0.53 [0.46, 0.59]***
Twitter + All$^{\dagger }$	0.63 [0.57, 0.68]***	0.54 [0.48, 0.60]***

Using measures recorded during the second wave of the opioid epidemic (2010–2012; heroin poisonings) to predict mortality in the third wave (2013–2019; synthetic poisonings).

***$${{p}} < 0.001$$.

**$${{p}} < 0.01$$.

*$${{p}} < 0.05$$.

$\dagger$out-of-sample Pearson correlation.

## Discussion

This study is the first to demonstrate the feasibility of predicting OPM by machine learning-based, natural language processing algorithms without relying on opioid-related keywords. We demonstrated that Twitter language-based models of OPM exceeds the predictive accuracy of widely used psychometric self-report measures and area-based covariates (Pearson’s r of 0.65 for language vs. 0.52, as seen in Fig. 2). They also provide further insights into communities suffering from large mortality rates. This method provides an efficient, unobtrusive, and cost-effective means by which to measure large-scale trends in the ongoing opioid epidemic. It is possible to leverage publicly available data sources with these methods, such as social media and search engine data, thereby providing a new public health tool capable of anticipating problem areas and potentially informing clinical efforts and public policy directives.

Unlike previous studies using machine learning-based methods to predict OPM from opioid keywords, the most predictive features of opiate use mortality do not include explicitly mention of drugs or drug use (e.g., Figs. 3, 4; see Supplementary Table S14 for full details on drug mentions). Instead, the linguistic features most predictive of OPM involve the expression of negative emotions, boredom, and work, while the linguistic features most negatively correlated with OPM involve positive emotions, growth, and leisure activities. These findings are consistent with previous work showing that community-level opioid mortality (e.g., state and metropolitan statistical areas) is predicted by low psychological well-being ^12^. These results can also be interpreted within the “deaths of despair” framing, which combines mortality from drug poisoning, alcohol liver disease, and suicide and posits that a decline in socioeconomic outlook is one factor driving increases in mortality  ^47–49^. While the deaths of despair framing is usually at the population-level, Shanahan et al. ^50^ define despair at the individual-level across four domains: cognitive (e.g., helplessness and limited positive expectations), emotional (e.g., sadness and loneliness), behavioral (e.g., risky and unhealthy acts), and biological (e.g., dysregulation or depletion). The results above include self-report and linguistic correlates associated with cognitive (low life satisfaction), emotional (negative emotions and sadness), behavioral (protective discussion of finances, travel, and hobbies), and biological despair (lack of sleep). Though these linguistic signals appear to be consistent with both theories of despair and deaths of despair, care must be taken in overgeneralizing these linguistic features.

The temporal analysis across Waves 2 and 3 (heroin vs synthetic opioids) shows that several variables did not significantly correlate with *changes* between the two waves, despite correlating with rates during each wave. All variables within the subjective well-being category remained significant when examining changes in mortality rates, whereas all socioeconomics, pain, depression, and 2 out of 5 demographics (percent white and percent rural) did not. Twitter language was able to predict changes in mortality rates, out-of-sample. It is important to note that the waves were defined nationally and that counties may have experienced increases in heroin (Wave 2) and synthetic opioids (Wave 3) at different times. While these results may suggest that social media data and psychometric self-reports can be used for a temporal analysis, further work is needed to verify this (e.g., predictions across each year).

The results presented here contribute to a line of work using digital sources to measure indicators of community distress ^51^. The Twitter language results in Figs. 3 and 4 tell a story of emotional pain and economic disadvantage (i.e., the Work/Long Hours/Dread category in Fig. 3 is predictive of OPM while Leisure/Travel category in Fig. 4 is protective). Similar structural factors of disadvantage and low physical and psychological well-being predict both future mortality and changes in mortality across the epidemic’s waves. Future work could example how these structural factors of disadvantage, as measured through digital data, vary across different spatial aggregations (such as Census regions or even within-city variations), socio-demographic populations, and temporal spans (e.g., opioid poisonings during COVID-19).

Given the ecological nature of the study, one must take care when interpreting these results due to ecological fallacies ^52^. The county-level correlations should not be used to infer relationships between individuals, just as individual-level correlations should not be used to infer relationships about counties (or any aggregate group). Similarly, as with all spatial-level analyses, the results may be dependent on the level of aggregation, which is known as the modifiable areal unit problem (MAUP) ^53^. Thus, one must not assume that results at the county-level hold for other spatial units, such as smaller, more heterogeneous zip codes or larger U.S. states.

Each data set brings along additional limitations. Due to privacy concerns, age-adjusted mortality rates are reported by the CDC only when the number of deaths is above 20. Thus, less populated counties may not be represented. The self-report data from Gallup often suffers from response biases ^54^, aggregated Twitter data suffers from selection biases (both in terms of Twitter itself, e.g., skews towards younger demographics, and through the county-mapping process, i.e., only considers user who self-disclose their location) ^55^, and both data sets suffer from spatial dependencies (i.e., counties close in space will often have similar outcome values ^56^). Using complementary heterogeneous data sets is one way to help mitigate these limitations.

Opiate mortality and declining psychological well-being have contributed to falling life expectancy in the U.S. Using heterogeneous data sources and machine learning-based, natural language processing methods, we have provided a method to predict opiate mortality risk that meets or exceeds psychometric self-report methods. Additionally, social media language correlates are in line with previous results at both the community and individual levels, showing that increased opioid mortality is associated with decreased psychological well-being. This study represents a significant advance in measurement methods available to researchers, clinicians, and public health officials concerned with the opioid epidemic or substance use, in general. Social media-based assessments are unobtrusive, low-cost, and can be applied at scale ^57^. Further, they offer the potential to deliver interventions in real-world settings ^58^. Thus, this language-based method provides a new tool for epidemiologists and public health researchers.

## Methods

### Data

A total of $n = 662$ counties met the opioid mortality, Gallup, Twitter, and area-based covariate data requirements (see Supplementary Figure S1 for inclusion criteria flow chart). These 662 counties represent 89.4% of all fatal opioid related poisonings across 2017 and 2018.

#### Opioid mortality

We collected county-level, age-adjusted opioid mortality data from the Centers for Disease Control and Prevention (CDC) WONDER online database from the years 2017 and 2018. We chose this time period so that the predictors (see below) were measured before the outcome (i.e., OPM). While this does not imply causation, it does enforce a temporal distance between the measurements (e.g., life satisfaction and language, a behavioral measure, are evaluated before OPM). The CDC censors age-adjusted mortality rates for counties with less than 20 deaths and crude rates (not age-adjusted) for counties with less than 10 deaths. In order to minimize the influence of age in our results, we used the age-adjusted rate and therefore did not consider counties with less than 20 deaths. See Supplemental Materials for a discussion on using non-age-adjusted rates. Fatal opioid poisonings are identified by the presence of any of the following multiple cause of death codes (ICD-10): opium, T40.0; heroin, T40.1; natural and semisynthetic opioids T40.2; methadone, T40.3; synthetic opioids, T40.4; or other and unspecified narcotics, T40.6. This resulted in $n = 724$ counties with mortality data. See Supplemental Materials for a discussion on underlying cause of death codes.

#### Psychometric self-reports

We defined physical and psychological well-being through four dimensions: experienced and evaluative subjective well-being ^59^, depression, and physical pain. These dimensions were measured via psychometric self-reports using the Gallup-Sharecare Well-Being Index, a large national longitudinal survey. Following Ward et al. ^60^, experienced subjective well-being was measured as positive affect (average response to happiness, enjoyment, and laughter) and negative affect (the average response to stress, worry, and sadness). Evaluative subjective well-being was measured via Cantril’s ladder (i.e., life satisfaction), which asks survey participants to evaluate their life as a whole, both today and five years from now. Depression was measured as the percentage of participants who have been told by a clinician that they have depression. Similarly, pain was measured as the percentage of participants who reported physical pain the day preceding the survey. Our sample only included people who responded to each of the above measures. See Supplementary Table S1 for full details on Gallup questions.

We used these person-level self-reports to obtain county-level averages. To match the time span of the Twitter data (see below), we only included self-reports from 2009 to 2015. Following Jaidka et al. ^61^, we set a minimum of 300 self-reports per county, resulting in 1,509,193 self-reports averaged to $n = 1059$ counties. Of these, $n = 664$ (1,302,830 self-reports) counties also had OPM data available. See Supplementary Table S2 for county-level statistics.

#### Twitter

Twitter data was taken from the County Tweet Lexical Bank (CTLB) ^62^, an open source data set of U.S. county-level language features. This data set is derived from a larger, random 10% sample of Twitter between 2009 and 2015, also known as the Decahose (see Giorgi et al.^62^ for full details on this data set.) While the Twitter API described this public stream as “random”, research has shown that this is not the case and opaque internal algorithms impact the types of data available via this stream^63^. The CTLB consists of over 1.5 billion tweets from roughly 6 million U.S. county-mapped Twitter users. Twitter users are mapped to U.S. counties through either latitude/longitude coordinates associated with their tweets or self-reported location information in the user’s profile. Latitude/longitude coordinates are trivially mapped to U.S. counties. The self-reported location information is extracted from a free text field in the user’s profile using a rule-based method which was designed to minimize false positives at the expense of a smaller number of mappings ^45^. The full set of county mapping rules can be found in Schwartz et al. ^45^. A total of 2041 counties were contained in this data set, each county with a minimum of 100 Twitter users, who, in turn, must have at least 30 tweets in the data set. Twitter language features are described below. After overlapping these 2041 counties with the 664 counties with psychometric self-reports we were left with $n = 662$ distinct counties. See Supplementary Table S2 for county-level statistics.

#### Area-based covariates

Following Woolf and Schoomaker ^46^, we considered a number of additional explanatory variables known to be related to OPM. This was done in order to establish a predictive baseline and includes demographics, socioeconomics, and access to health care. Supplementary Table S5 contains additional categories of variables related to behavioral health measures, pharmacotherapy access ^64^, income inequality, and racial segregation. The demographic category includes the percentage female, percentage white, percentage over 65 years of age, median age, and percentage of the population living in rural communities. Socioeconomic variables include the percentage of the population with at least a high school diploma, percentage of the population with at least a Bachelor’s degree, median household income (logged to prevent skewness), and unemployment rates. Access to health care includes the number of primary care physicians, the number of mental health providers, and the percentage of the population with insurance. When available, all variables were collected from the same time span as the well-being and Twitter data (i.e., 2009–2015; see Supplementary Table S3 for full detail on data sources).

### Statistical analysis

All analyses were performed using the Differential Language Analysis ToolKit (DLATK) open-source Python package ^65^.

#### Predictive baseline via psychometric self-reports and area-based covariates

We examined the relationship between opioid mortality with psychometric self-reports of physical and psychological well-being by first considering each variable independently and then considering categories of predictors. For each variable (e.g., physical and psychological well-being and U.S. Census variables), we computed a Pearson correlation with OPM. To correct for multiple comparisons, we used a Benjamini–Hochberg False Discovery Rate correction ^66^, with a statistically significant level at ${{p}} <.05$. Since Pearson correlations measure linear relationships, we visualize each relationship as a scatter plot and provide Spearman correlation values in Figure S2 in the Supplemental Materials.

To evaluate the predictive baselines of categories of variables, we used a multiple regression where OPM was the dependent variables and all variables within each category (e.g., access to health care) were independent variables. We used a tenfold cross-validation setup for out-of-sample evaluation. We randomly split all counties into 10 mutually exclusive chunks (or folds), trained a linear regression on 9 of the folds, and then evaluated the model on the held-out 10th fold. We repeated this process ten times, such that each fold was used for evaluation exactly once. We then took the out-of-sample prediction values and correlated them with the ground truth (i.e., CDC opioid mortality rates). In order to access the upper bound on the prediction accuracy, we ran tenfold cross-validation using a model with all physical and psychological well-being and area-based measures.

#### Predictive accuracy of Twitter

We repeated the above analysis using county-level language estimates derived from Twitter, available via the County Tweet Lexical Bank. Each county was represented as a collection of 25,000 word (or unigram) frequencies, where the word frequencies are first calculated for each user in the data set and then averaged across all users within each county. The word frequencies are then used to derive 2000 Latent Dirichlet Allocation ^67^ (LDA) topic frequencies per county, using an open-source set of LDA topics previously estimated over a large corpus of approximately 15 million Facebook statuses ^68^. These topics have previously been used to study a number of constructs at the community level including heart disease and excessive drinking ^41,42^. The probability of topic usage per county was estimated as

1 $$\begin{aligned} P\big (\text {topic}\big |\text {county}\big ) = \sum _{\text {word}\in \text {topic}}P\big (\text {topic}\big |\text {word}\big )\times P\big (\text {word}\big |\text {county}\big ). \end{aligned}$$

Here $P\big (\text {word}\big |\text {county}\big )$ is the probability of the word given the county (estimated using the word frequency) and $P\big (\text {topic}\big |\text {word}\big )$ is the probability of the topic given the word (estimated from the LDA process).

To predict opioid mortality rates from the 2000 county LDA topics, we used a pipeline of feature selection, principal component analysis (PCA), and Ridge regression (i.e., an $l_2$ penalized linear regression). The feature selection pipeline first removed all low variance features. Next, PCA was applied to reduce the dimension of the feature space in order to avoid overfitting, since the number of features (2000 topics) is larger than the number of observations (662 counties). Finally, the PCA reduced topics were then used as features in a Ridge regression. In order to assess out-of-sample accuracy, we used tenfold cross validation, as described above, correlating the out-of-sample predictions with the ground truth mortality rates (Supplemental Materials Table S13 contains additional metrics such as mean absolute error and mean squared error). The regularization term $\lambda$ in the Ridge regression was chosen via the cross validation process. Finally, we pair-wise compare feature sets using a paired t-test on each model’s absolute error to access if one model’s accuracy is significantly greater than another’s.

#### Linguistic insights

After establishing a baseline predictive accuracy, we examined what these communities were discussing on social media. To do this, we used a process called Differential Language Analysis (DLA) ^68^. For each of the 2000 topics described above, we performed a linear regression where the county-level topic loading and OPM were the independent and dependent variables, respectively. Socio-demographics variables were included as covariates in each regression and all variables were mean-centered and normalized by their respective standard deviation. Given the large number of comparisons (i.e., 2000), we applied a Benjamini–Hochberg False Discovery Rate correction, with a statistically significant level of ${{p}} <.05$. We visualized the significant topics as a word cloud.

#### Temporal trends in opioid mortality

Finally, we examined temporal trends in OPM. The CDC defined three waves across the opioid epidemic: (1) a rise in prescription opioid poisonings starting in 1999, (2) a rise in heroin poisonings starting in 2010, and (3) a rise in synthetic opioid poisonings starting in 2013. We looked at how psychometric self-reports, social media lanugage, and area-based covariates *as measured during the second wave* (i.e., 2010 to 2012) predicts mortality *during the third wave* (2013 to 2019), as well as changes in mortality between Waves 2 and 3. Due to lack of historic data we are unable to examine Wave 1. To do this, we aggregated Gallup responses and Twitter data during Wave 2 and correlated the county averages with both Wave 3 mortality rates and the difference in mortality between Wave 2 and Wave 3.

In order to temporally align our predictor variables (e.g., Gallup psychometrics self-reports and Twitter data) with OPM during Wave 2, we limited our data to the years 2010 to 2012. Using the same thresholds as above (counties with at least 300 Gallup responses and 100 Twitter users, each with 30 or more tweets), we aggregated 726,301 Gallup responses and 381,470,253 tweets from 2,219,131 Twitter users across 2010–2012. A total of 522 U.S. counties met the required thresholds.

### Ethics statement

This study has been reviewed and approved by an academic Institutional Review Board and deemed exempt (Category 4; secondary research). No humans were directly involved in this study. We use the term “poisoning” instead of “overdose” to avoid the stigma associated with “overdose”, i.e., implications that (1) there is a correct and safe dose and that (2) the substance user knows what a proper dose is and chose to take more. We also note that “poisoning” more accurately reflects the diagnosis term of what is happening in the body clinically.

## Supplementary Information

Supplementary Information.

## Data Availability

All data used in this study, with the exception of the Gallup data, are collected from publicly available sources. As such, we have made available all aggregated data used in this study: https://osf.io/dnejr/. All data, including Twitter, is aggregated and anonymized. No individual-level estimates or intermediate data will be made available. Due to Twitter’s Terms of Service, individual tweets will not be made available.
