# Supplementary material for: Predicting U.S. county opioid poisoning mortality from multi-modal social media and psychological self-report data
Source: Sci Rep. 2023 Jun 3;13:9027. doi: 10.1038/s41598-023-34468-2 (PMC10238775; doi:10.1038/s41598-023-34468-2)
Supplement: Supplementary file 1 — Supplementary Information. [file 41598_2023_34468_MOESM1_ESM.pdf]

Supplementary Material for "Predicting U.S. County Opioid Poisoning Mortality From Multi-Modal Social Media and Psychological Self-Report Data"

Salvatore Giorgi[1,2], David B. Yaden[3], Johannes C. Eichstaedt[4,5], Lyle H. Ungar[2], H. Andrew Schwartz[6], Amy Kwarteng[1], and Brenda Curtis[1,*]

[1]Intramural Research Program, National Institute on Drug Abuse
[2]Department of Computer and Information Science, University of Pennsylvania
[3]Department of Psychiatry and Behavioral Sciences, Johns Hopkins University School of Medicine
[4]Department of Psychology, Stanford University
[5]Institute for Human-Centered AI, Stanford University
[6]Department of Computer Science, Stony Brook University
*Corresponding author: brenda.curtis@nih.gov

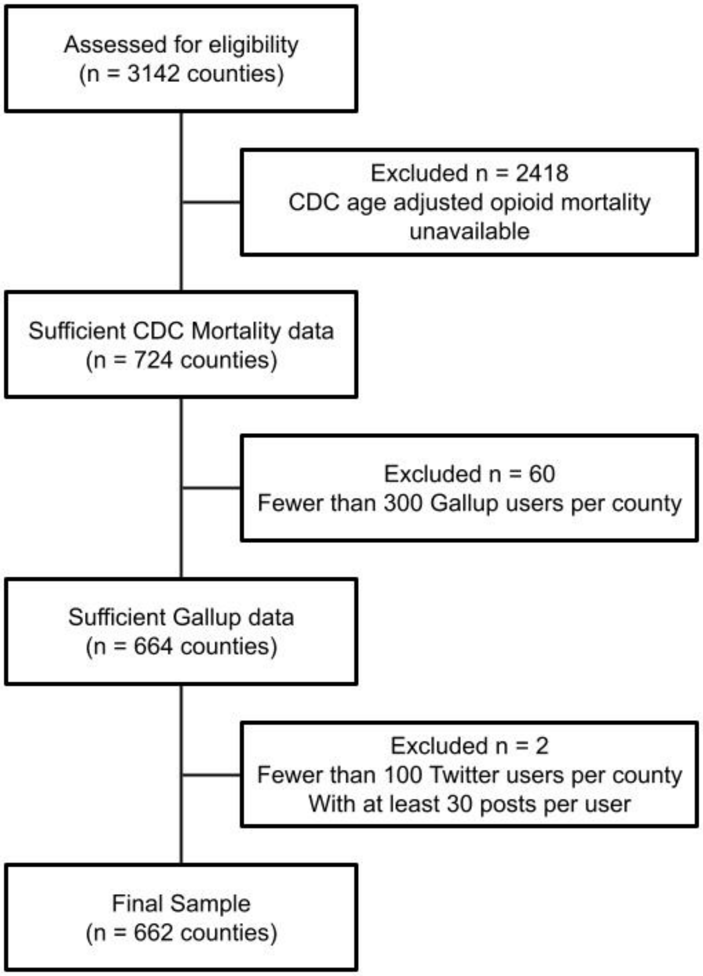

Figure S1: County level inclusion criteria

Item	Question	Scale	Mean (SD)
Depression	Have you ever been told by a physician or nurse that you have any of the following, or not? How about depression?	0-1	0.16 (0.36)
	Please imagine a ladder with steps numbered from zero at the bottom to ten at the top. The top of the ladder represents the best possible life for you and the bottom of the ladder represents the worst possible life for you.		
LS today	On which step of the ladder would you say you personally feel you stand at this time?	0-10	7.09 (1.89)
LS five years	On which step do you think you will stand about five years from now?	0-10	7.61 (2.25)
Smile	Did you smile or laugh a lot yesterday?	0-1	0.83 (0.38)
	Did you experience the following feelings during A LOT OF THE DAY yesterday? How about:		
Happy	Happiness	0-1	0.89 (0.31)
Enjoyment	Enjoyment	0-1	0.86 (0.35)
Sad	Sadness	0-1	0.16 (0.37)
Worried	Worry	0-1	0.29 (0.45)
Stress	Stress	0-1	0.37 (0.48)
Pain	Physical pain	0-1	0.23 (0.42)

Table S1: Summary statistics of Gallup variables

	Min	Max	Mean (SD)	Total
Gallup Responses	300	35295	1966.4 (2579.9)	1,301,734
Twitter Users	116	394490	7926.8 (23099.2)	5,247,530

Table S2: County level summary statistics

Variable	Category	Source	Years	Description
% Rural	Demographics	Census Population Estimates, obtained through County Health Rankings (CHR) 2015	2010	Percentage of the population living in a rural area
% Over 65		Census Population Estimates, obtained through County Health Rankings (CHR) 2017	2015	Percentage of the population 65 years of age or older
% Female		American Community Survey (ACS) 2014, 5 year estimates	2014	Percentage of the female population
% White				Percentage of the Non-Hispanic white population
Median Age				Median age
Median Income	Socioeconomics			Median household income (log transformed)
Unemployment Rate				Percentage of the population reported as unemployed
% with a Bachelor's Degree				Percentage of population with a Bachelor's degree or higher
% with a High School diploma				Percentage of population with a high school diploma (or equivalent) or higher
% Uninsured	Access to Health Care			Percentage of the population without insurance
Mental Health Providers		CMS, National Provider Identification file, obtained through County Health Rankings (CHR) 2015	2014	Ratio of population to mental health providers
Primary Care Physicians		Area Health Resource File/American Medical Association, obtained through County Health Rankings (CHR) 2015	2012	Ratio of population to primary care physicians
Smoking	Health Behaviors	Behavioral Risk Factor Surveillance System, obtained through County Health Rankings (CHR) 2015	2006-2012	Percentage of adults who are current smokers
Obese		CDC Diabetes Interactive Atlas, obtained through County Health Rankings (CHR) 2015	2011	Percentage of adults that report a BMI of 30 or more
Physicians Licensed to Administer Buprenorphine	Pharmacotherapy Access		2017	Number of physicians authorized to treat opioid dependency with buprenorphine by state
Facilities Providing All Medication Assisted Treatments		The National Survey of Substance Abuse Treatment Facilities (N-SSATS)	2017	Number of substance abuse treatment facilities offering all three Medication Assisted Treatments services (Buprenorphine, Methadone, Naltrexone)
Good Samaritan Law		Prescription Drug Abuse Policy System (PDAPS)	2017	Good Samaritan laws provide immunity from arrest, charge, or prosecution for controlled substance possession when seeking medical assistance for opioid related overdoses. State-level binary indicator.
Naloxone prescribers			2017	State laws which provide immunity to individuals who administer, possess, or prescibe (in the case of medical professionals) naloxone. State-level binary indicator.
Segregation Index	Economic and Racial Inequality	American Community Survey, obtains through County Health Rankings (CHR) 2016	2010-2014	Represents the percent of Black residents that would need to relocate to be fully integrated with white residents across metropolitan neighborhoods
Gini Income Inequality		American Community Survey (ACS) 2014, 5 year estimates	2014	Measures the distribution of income across the population, with higher values representing more inequality.

Table S3: Data sources for all area-based covariates

		Perc. Female	Perc. White	Perc. Rural	Perc . Over 65	Median Age	Perc. w/ HS Diploma	Unemploy. Rate	Med. Inc.	Perc. w/ Bach Degree	Mental Health Prov.	Primary Care Prov.	Perc. Insured	Pos. Emo.	Neg. Emo.	Life Sat. (5 years)	Life Sat. (today)	Depress.	Pain	Perc. Adult Smokers	Perc. Adult Obesity	Bup. Physic.	Nalox. Prescrib.	Good Sam Law	Med. Assisted Treat.	Gini Income Inequal.	Racial Seg.
Demographics	Perc. Female	1																									
	Perc. White	-0.29	1																								
	Perc. Rural	-0.25	0.50	1																							
	Perc. Over 65	0.06	0.40	0.37	1																						
	Median Age	0.07	0.44	0.44	0.90	1																					
Socio-economics	Perc. w/ HS Diploma	-0.01	0.25	-0.15	0.01	0.11	1																				
	Unemployment Rate	0.07	-0.33	0.04	0.19	0.07	-0.55	1																			
	Median Income	-0.07	-0.05	-0.33	-0.30	-0.08	0.54	-0.53	1																		
	Perc. w/ Bach Degree	0.17	-0.22	-0.50	-0.30	-0.23	0.57	-0.49	0.70	1																	
Access to Healthcare	Mental Health Prov.	0.14	-0.11	-0.20	-0.05	-0.08	0.21	-0.07	0.12	0.44	1																
	Primary Care Prov.	0.29	-0.13	-0.33	-0.09	-0.07	0.35	-0.30	0.24	0.63	0.58	1															
	Perc. Insured	0.02	-0.33	-0.10	-0.05	-0.21	-0.65	0.45	-0.45	-0.33	-0.19	-0.24	1														
Subjective Well-being	Pos. Emo.	-0.02	-0.01	-0.21	-0.06	-0.13	0.38	-0.33	0.32	0.36	0.02	0.14	-0.06	1													
	Neg. Emo.	0.04	0.04	0.08	-0.15	-0.10	-0.34	0.20	-0.19	-0.13	0.14	0.00	0.10	-0.67	1												
	Life Sat. (5 years)	0.17	-0.67	-0.62	-0.61	-0.60	0.15	-0.13	0.45	0.61	0.23	0.29	0.10	0.35	-0.17	1											
	Life Sat. (today)	0.08	-0.21	-0.35	-0.12	-0.20	0.32	-0.40	0.46	0.63	0.21	0.36	-0.07	0.64	-0.49	0.62	1										
Depression	Depression	-0.04	0.29	0.42	0.14	0.10	-0.35	0.24	-0.63	-0.50	0.08	-0.09	0.14	-0.49	0.50	-0.51	-0.48	1									
Pain	Pain	-0.11	0.32	0.49	0.34	0.29	-0.41	0.42	-0.66	-0.74	-0.17	-0.34	0.22	-0.52	0.43	-0.68	-0.62	0.77	1								
Behavioral Health	Perc. Adult Smokers	-0.01	0.21	0.44	0.25	0.25	-0.32	0.34	-0.62	-0.71	-0.32	-0.39	0.15	-0.41	0.19	-0.55	-0.62	0.55	0.66	1							
	Perc. Adult Obesity	0.03	0.04	0.37	0.02	0.02	-0.31	0.27	-0.51	-0.70	-0.45	-0.41	0.12	-0.26	0.04	-0.36	-0.46	0.42	0.52	0.64	1						
Pharmaco-therapy Access	Buprenorphine Physicians	0.22	-0.39	-0.43	-0.15	-0.14	-0.08	0.06	0.16	0.33	0.32	0.30	0.06	-0.11	0.19	0.36	0.10	-0.17	-0.26	-0.28	-0.34	1					
	Naloxone Prescribers	-0.06	-0.02	-0.02	-0.02	0.01	-0.01	0.00	0.09	-0.01	-0.09	-0.06	-0.07	-0.02	-0.01	0.01	-0.04	-0.14	-0.11	-0.04	-0.06	0.05	1				
	Good Sam Law	0.04	-0.01	0.04	0.17	0.17	0.10	0.17	0.00	-0.02	-0.04	0.00	-0.26	-0.09	0.04	-0.13	-0.14	-0.01	0.07	0.07	-0.04	0.00	0.12	1			
	Med. Assisted Treatment	0.18	-0.27	-0.29	-0.10	-0.09	-0.04	0.05	0.12	0.25	0.29	0.23	-0.05	-0.10	0.14	0.24	0.05	-0.10	-0.19	-0.19	-0.25	0.72	-0.01	0.07	1		
Inequality	Gini Income Inequality	0.32	-0.38	-0.32	0.03	-0.11	-0.23	0.23	-0.28	0.27	0.40	0.47	0.31	-0.09	0.20	0.24	0.17	0.05	-0.09	-0.12	-0.27	0.44	-0.08	-0.04	0.32	1	
	Racial Segregation	0.03	0.17	0.00	0.18	0.18	0.05	-0.02	-0.16	-0.07	0.18	0.16	-0.14	-0.14	0.16	-0.17	-0.16	0.15	0.14	0.05	-0.04	0.19	0.06	0.04	0.16	0.22	1

Table S4: Pearson correlations between all psychometric self-reports and area-based covariates.

	Correlation with OPM
Behavioral Health	0.37
Perc. Adult Smokers	0.38
Perc. Adult Obesity	0.25
Pharmacotherapy Access	0.15
Good Sam Law	0.17
Naloxone Prescribers	-0.08
Buprenorphine Physicians	-0.02
Medication Assisted Treatment	0.06
Inequality	0.15
Racial Segregation	0.17
Gini Income Inequality	0.00

Table S5: Correlation between OPM and behavioral health, pharmacotherapy access, and inequality measures. Reported in-sample Pearson correlation except for groups of predictors (italicized), which uses a 10-fold cross validation setup.

	Opioid Mortality					
	(1)	(2)	(3)	(4)	(5)	(6)
Positive Emotions	-0.32*** (0.04)					
Negative Emotions		0.29*** (0.04)				
Life Satisfaction (today)			-0.31*** (0.04)			
Life Satisfaction (5 years)				-0.31*** (0.06)		
Depression					0.28*** (0.04)	
Pain						0.27*** (0.04)
Perc. Female	0.18*** (0.04)	0.15*** (0.04)	0.19*** (0.04)	0.19*** (0.04)	0.15*** (0.04)	0.18*** (0.04)
Perc. White	0.16*** (0.04)	0.08 (0.04)	0.10* (0.04)	-0.01 (0.05)	0.06 (0.04)	0.09* (0.04)
Perc. Rural	0.05 (0.04)	0.09* (0.04)	0.04 (0.04)	0.04 (0.05)	0.00 (0.05)	0.01 (0.05)
Median Age	0.22** (0.08)	0.32*** (0.08)	0.25** (0.08)	0.33*** (0.08)	0.47*** (0.08)	0.42*** (0.08)
Perc. Over 65	-0.05 (0.08)	-0.05 (0.08)	-0.06 (0.08)	-0.25** (0.08)	-0.23** (0.08)	-0.26** (0.08)
Counties	662	662	662	662	662	662
R^2	.24	.23	.23	.18	.21	.20

Table S6: Gallup psychological well-being measures with Demographic controls. Reported standardized betas and standard errors. *** $p < 0.001$, ** $p < 0.01$, * $p < 0.05$

	Opioid Mortality					
	(1)	(2)	(3)	(4)	(5)	(6)
Positive Emotions	-0.37*** (0.04)					
Negative Emotions		0.33*** (0.04)				
Life Satisfaction (today)			-0.37*** (0.05)			
Life Satisfaction (5 years)				-0.32*** (0.05)		
Depression					0.30*** (0.05)	
Pain						0.33*** (0.06)
Perc. High School	0.23*** (0.05)	0.28*** (0.05)	0.11* (0.05)	0.07 (0.05)	0.17*** (0.05)	0.13** (0.05)
Perc. Bach. Degree	-0.18*** (0.05)	-0.29*** (0.05)	-0.01 (0.06)	-0.01 (0.07)	-0.20*** (0.06)	-0.06 (0.06)
Median Income (log)	-0.09 (0.05)	-0.07 (0.05)	-0.10 (0.05)	-0.04 (0.05)	0.08 (0.06)	-0.00 (0.06)
Unemployment rate	-0.04 (0.05)	-0.01 (0.05)	-0.05 (0.05)	0.06 (0.05)	0.06 (0.05)	-0.00 (0.05)
Counties	662	662	662	662	662	662
R^2	.18	.16	.15	.12	.12	.11

Table S7: Gallup psychological well-being measures with Socioeconomic controls. Reported standardized betas and standard errors. *** $p < 0.001$, ** $p < 0.01$, * $p < 0.05$

	Opioid Mortality					
	(1)	(2)	(3)	(4)	(5)	(6)
Positive Emotions	-0.37*** (0.04)					
Negative Emotions		0.32*** (0.04)				
Life Satisfaction (today)			-0.39*** (0.04)			
Life Satisfaction (5 years)				-0.33*** (0.04)		
Depression					0.35*** (0.04)	
Pain						0.38*** (0.04)
Primary Care Providers	-0.05 (0.04)	-0.09 (0.04)	0.02 (0.05)	-0.02 (0.05)	-0.06 (0.04)	0.01 (0.05)
Mental Heath Providers	-0.02 (0.04)	-0.06 (0.05)	0.01 (0.04)	0.04 (0.04)	-0.06 (0.04)	-0.01 (0.04)
Uninsured	-0.24*** (0.04)	-0.27*** (0.04)	-0.22*** (0.04)	-0.17*** (0.04)	-0.28*** (0.04)	-0.29*** (0.04)
Counties	662	662	662	662	662	662
R^2	.19	.15	.19	.15	.17	.18

Table S8: Gallup psychological well-being measures with Access to Health Care controls. Reported standardized betas and standard errors. *** $p < 0.001$, ** $p < 0.01$, * $p < 0.05$

	Opioid Mortality					
	(1)	(2)	(3)	(4)	(5)	(6)
Positive Emotions	-0.35*** (0.04)					
Negative Emotions		0.29*** (0.04)				
Life Satisfaction (today)			-0.35*** (0.04)			
Life Satisfaction (5 years)				-0.37*** (0.04)		
Depression					0.31*** (0.04)	
Pain						0.32*** (0.04)
Good Sam Law	0.14*** (0.04)	0.16*** (0.04)	0.12*** (0.04)	0.12** (0.04)	0.17*** (0.04)	0.14*** (0.04)
Naloxone Prescribers	-0.09** (0.04)	-0.08* (0.04)	-0.10** (0.04)	-0.09* (0.04)	-0.05 (0.04)	-0.06 (0.04)
Buprenorphine Physicians	-0.13* (0.05)	-0.15** (0.05)	-0.06 (0.05)	0.03 (0.05)	-0.04 (0.05)	-0.03 (0.05)
Medication Assisted Treatment	0.11* (0.05)	0.12* (0.05)	0.11* (0.05)	0.12* (0.05)	0.11* (0.05)	0.13* (0.05)
Counties	662	662	662	662	662	662
R^2	.17	.12	.17	.16	.13	.14

Table S9: Gallup psychological well-being measures with Pharmacotherapy Access controls. Reported standardized betas and standard errors. *** $p < 0.001$, ** $p < 0.01$, * $p < 0.05$

	Opioid Mortality					
	(1)	(2)	(3)	(4)	(5)	(6)
Positive Emotions	-0.25*** (0.04)					
Negative Emotions		0.22*** (0.04)				
Life Satisfaction (today)			-0.21*** (0.05)			
Life Satisfaction (5 years)				-0.19*** (0.04)		
Depression					0.14** (0.04)	
Pain						0.12* (0.05)
Smoking	0.27*** (0.05)	0.31*** (0.05)	0.26*** (0.05)	0.27*** (0.05)	0.31*** (0.05)	0.31*** (0.05)
Obese	0.01 (0.05)	0.04 (0.05)	-0.01 (0.05)	0.01 (0.05)	-0.01 (0.05)	-0.01 (0.05)
Counties	662	662	662	662	662	662
R^2	.20	.19	.17	.17	.16	.16

Table S10: Gallup psychological well-being measures with Health behaviors controls. Reported standardized betas and standard errors. *** $p < 0.001$, ** $p < 0.01$, * $p < 0.05$

	Opioid Mortality					
	(1)	(2)	(3)	(4)	(5)	(6)
Positive Emotions	-0.34*** (0.04)					
Negative Emotions		0.26*** (0.04)				
Life Satisfaction (today)			-0.35*** (0.04)			
Life Satisfaction (5 years)				-0.32*** (0.04)		
Depression					0.26*** (0.04)	
Pain						0.27*** (0.04)
Segregration	0.13*** (0.04)	0.14*** (0.04)	0.10** (0.04)	0.10* (0.04)	0.13*** (0.04)	0.13*** (0.04)
Gini income inequality	-0.07 (0.04)	-0.09* (0.04)	0.03 (0.04)	0.05 (0.04)	-0.05 (0.04)	-0.01 (0.04)
Counties	652	652	652	652	652	652
R^2	.15	.09	.15	.13	.10	.10

Table S11: Gallup psychological well-being measures with Economic and Racial Inequality controls. Reported standardized betas and standard errors. *** $p < 0.001$, ** $p < 0.01$, * $p < 0.05$

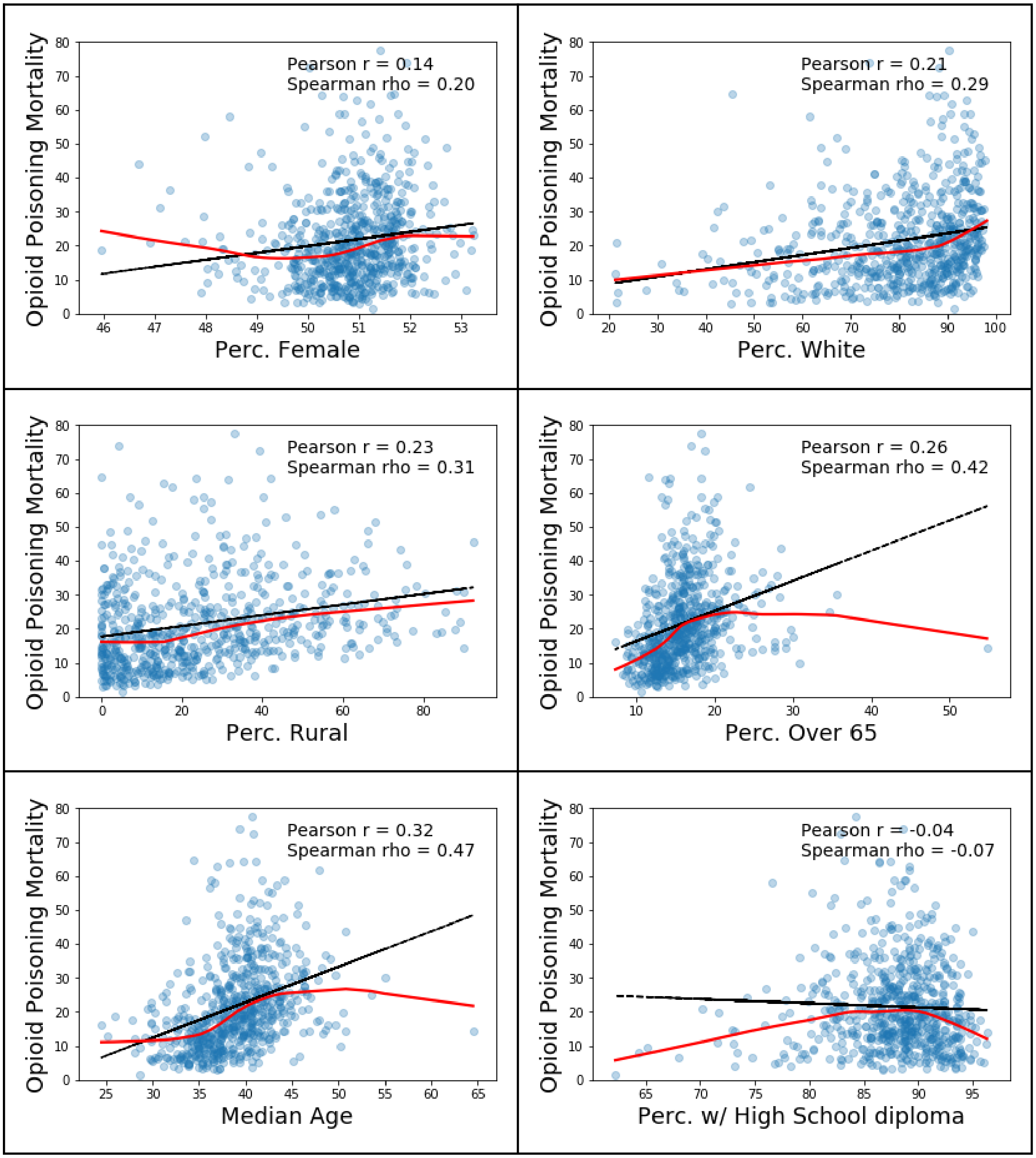
Pearson r = 0.14
Spearman rho = 0.20
Opioid Poisoning Mortality
Perc. Female
Pearson r = 0.21
Spearman rho = 0.29
Opioid Poisoning Mortality
Perc. White
Pearson r = 0.23
Spearman rho = 0.31
Opioid Poisoning Mortality
Perc. Rural
Pearson r = 0.26
Spearman rho = 0.42
Opioid Poisoning Mortality
Perc. Over 65
Pearson r = 0.32
Spearman rho = 0.47
Opioid Poisoning Mortality
Median Age
Pearson r = -0.04
Spearman rho = -0.07
Opioid Poisoning Mortality
Perc. w/ High School diploma

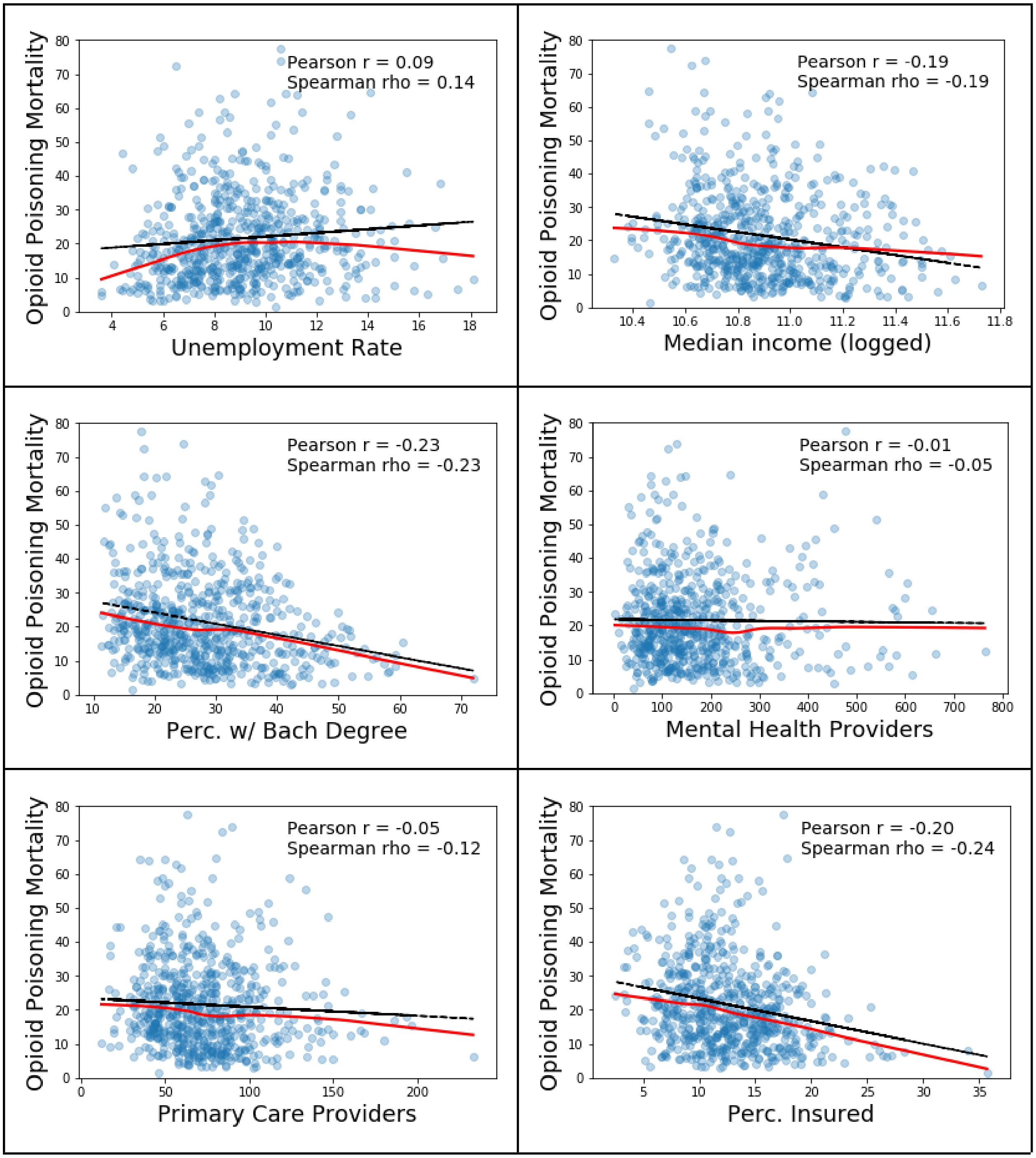

Pearson r = 0.09
Spearman rho = 0.14
Opioid Poisoning Mortality
Unemployment Rate
Pearson r = -0.19
Spearman rho = -0.19
Opioid Poisoning Mortality
Median income (logged)
Pearson r = -0.23
Spearman rho = -0.23
Opioid Poisoning Mortality
Perc. w/ Bach Degree
Pearson r = -0.01
Spearman rho = -0.05
Opioid Poisoning Mortality
Mental Health Providers
Pearson r = -0.05
Spearman rho = -0.12
Opioid Poisoning Mortality
Primary Care Providers
Pearson r = -0.20
Spearman rho = -0.24
Opioid Poisoning Mortality
Perc. Insured

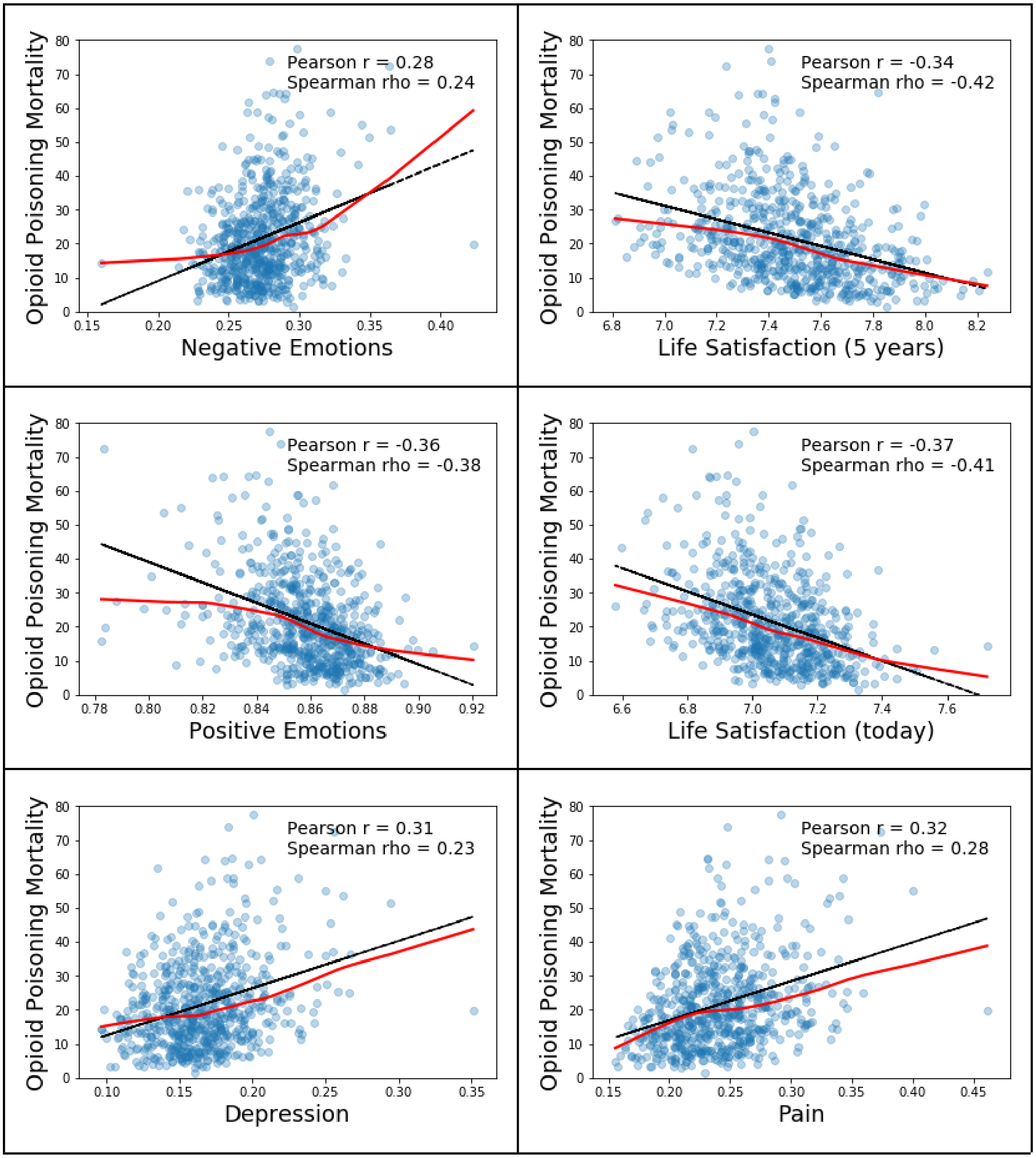

Figure S2: Scatter plots for all area-based covariates and psychometric self-reports. We report both Pearson and Spearman correlations, as well as linear fit lines (black) and lowess curve (red) in order to highlight both linear and non-linear relationships.

	Mean Squared Error	Mean Absolute Error
Twitter + All non-language	111.8	7.1
Twitter Alone	119.1	7.4
All non-language	148.9	8.5
Subjective Well-being	154.1	8.6
Access to Health Care	161.1	8.9
Depression	166.6	9.2
Pain	169.2	9.2
Socio-demographics	171.1	9.2

Table S12: Additional out-of-sample prediction metrics for all models evaluated in Figure 2. Mean Absolute Error shows that the Twitter + All non-language model is able to predict county-level opioid poisoning rates within 7.1 age-adjusted deaths per 100,000 people (on average).

Opioid Poisoning Mortality: Age-adjusted vs. Crude Rate

The CDC censors age-adjusted mortality rates for counties with less than 20 deaths and crude rates (not age-adjusted) for counties with less than 10 deaths. Thus, we could expand our sample size by considering the crude rate as opposed to the age-adjusted rate used throughout the manuscript. Using the same multiple cause of death codes, we collect non-age adjusted opioid poisoning mortality for 2017 and 2018. This is available for 921 counties which also have all other data available (Twitter, psychometric self-reports, and area-based covariates).

Since we would still like to minimize the influence of age in our results, we create an age-residualized version of the opioid mortality crude rate. To do this we gather national-level Census data from the 2014 American Community Survey. We collect the county-level percentage of the population in three age terciles (where the terciles are calculated at the national-level): younger than 25 years old, 25 to 49, and 50 years and older. We create a linear regression model where we predict OPM from the age terciles. We then use the residuals from this model as our age-adjust crude OPM rate. Finally, we predict the age-adjust crude OPM rate from our multimodal data (reproducing Figure 2) on the larger sample size (n = 921) and the sample used in the paper (n = 622). We also report the out-of-sample prediction accuracy using the CDC age-adjusted rate (i.e., the values from Figure 2) in order to aid the comparison. Here we note that we are changing both the sample size and the OPM measure. We consider both cases separately below.

We consider the manually age-adjusted OPM rate across the smaller sample size (i.e., the same sample size used throughout the main analyses) and compare this to the results in Figure 2, which uses the CDC age-adjusted mortality rate. As shown in the last two columns of Table S13, the CDC age-adjusted mortality rate is easier to predict from both Twitter and non-language variables. One possible explanation for this is that the residualizing process may be a stricter age-adjustment process and, therefore, removes any age signal from the outcome. Thus, any age-related features (e.g., median age, percentage of the population over 65, and Twitter language) will be weaker predictors.

Comparing the first two columns of Table S13 (i.e., comparing changes in sample sizes as opposed to comparing changes in age-adjustments), we see that the large sample size has (1) smaller predictive accuracy when using Twitter-based models and (2) little or no change in predictive accuracy when using non-Twitter-based models (the area-based covariates and psychometric self-reports). We note that the additional 259 counties used in the expanded sample size have a much smaller number of Twitter users making up the language estimates. The average number of Twitter users per county in the 622 counties is 7,926.8 (SD = 23,099.2; median = 2,091), whereas the average in the 259 additional counties is 1052.4 (SD = 1,265.1; median = 1,410). It could be that the language estimates for the 259 are noisier than the 622 counties, due to the smaller number of observations that are being aggregated. Past research has shown that a larger number of Twitter users per county results in higher out-of-sample prediction accuracy (Giorgi et al., 2018).

	Manual Age-adjustment		CDC Age-adjustment
Twitter + All non-language	0.57	0.63	0.68
Twitter Alone	0.54	0.59	0.65
All non-language	0.44	0.45	0.52
Subjective Well-being	0.41	0.43	0.49
Access to Health Care	0.37	0.38	0.46
Depression	0.34	0.37	0.43
Pain	0.33	0.34	0.41
Socio-demographics	0.31	0.32	0.40
n	921	622	662

Table S13: Out-of-sample prediction accuracy using the age-adjusted OPM rate across a larger sample (n = 921) and the sample used in the paper (n = 622). We include the results from Figure 2 in the column CDC age-adjustment to aid the comparisons.

Underlying and Multiple Cause of Death Codes

The opioid poisoning mortality rates used in this work were collected from the CDC WONDER database using the multiple cause of death codes without considering the underlying cause of death. To address this we obtained age-adjusted rates using the multiple cause of death ICD-10 codes (T40.0, T40.1, T40.2, T40.3, T40.4, and T40.6) along with the following underlying cause of death codes: X40-44, X60-64, X85, and Y10-Y14. We then correlated these rates with rates used in the main analysis. The two rates correlated at Pearson r = 0.99.

Drug Lexical Analysis

Rather than looking for general insights into these communities, we would like to know if communities who suffer from higher opioid poisonings also talk more about drugs and/or alcohol. We used several substance use related lexica with categories for alcohol, drugs, smoking and recovery. For each lexica, we count the number of words appearing in the respective lexical category (e.g., *drugs*, *smoking*, or *heroin*) and normalize that count by the total number of words

written by each county. We then correlate, at the county-level, the normalized word count with OPM.

We first used the 2018 list of Slang Terms and Code Words from the Drug Enforcement Administration (DEA)[2] and consider the *fentanyl*, *heroin*, and *all* categories. Note that the most frequent words in our data set are ambiguous e.g. "facebook" is defined by the DEA as "fentanyl mixed with heroin in pill form." The next substance use related lexicon we used has three categories, each related to various types of substance use (alcohol, drugs, and smoking), and a single category containing all words from the three smaller categories[3]. These categories were derived from a large sample of substance abuse related tweets and used to investigate relationships between substance abuse and various socio-demographic variables at the community level (i.e., U.S. zip codes). Finally, another study attempted to identify youth beliefs and behaviors in relation to drug use through a sample of tweets from young adults geotagged in Pennsylvania[4]. In general, these lexica tend to use highly specific words or phrases. Due to their infrequent nature, they do not appear in our Twitter data set, which consists of 25,000 unigrams (the most frequent in the data set).

Substance Category	Most Frequent Words	Correlation with OPM
Drug Enforcement Administration (DEA)		
Fentanyl	girl, friend, crazy, food, facebook	0.14
Heroin	night, down, him, girl, black	0.20
All	up, love, go, day, night	0.22
Meng et al., 2017		
Alcohol	drunk, beer, alcohol, vodka	0.07
Drugs	get high, cocaine, smoke weed, smokir	0.09
Smoking	beer, #beer, tobacco, cigars	0.03
All	drunk, beer, alcohol, vodka, get drunk	0.07
Stevens et al., 2019		
Drugs	high, drunk, smoke, beer, roll	0.00

Table S14: Pearson correlation with substance related lexica along with the top five most frequent words from each lexica appearing in our Twitter data set.

References

1. Giorgi, S., Preoţiuc-Pietro, D., Buffone, A., Rieman, D., Ungar, L., & Schwartz, H. A. The Remarkable Benefit of User-Level Aggregation for Lexical-based Population-Level Predictions. In Proceedings of the 2018 Conference on Empirical Methods in Natural Language Processing (2018).
2. Drug Enforcement Administration. Slang terms and code words: A reference for law enforcement personnel. DEA Intell. Rep. DEAHOU-DIR-022 18, 2018–07 (2018).
3. Meng, H.-W., Kath, S., Li, D. & Nguyen, Q. C. National substance use patterns on twitter. PLoS One 12, e0187691 (2017).
4. Stevens, R. C. et al. Exploring substance use tweets of youth in the united states: Mixed methods study. JMIR Public Heal. Surveill 6, e16191, DOI: 10.2196/16191 (2020).
